# Supplementary material for: Mechanisms governing the pioneering and redistribution capabilities of the non-classical pioneer PU.1
Source: Nat Commun. 2020 Jan 21;11:402. doi: 10.1038/s41467-019-13960-2 (PMC6972792; doi:10.1038/s41467-019-13960-2)
Supplement: Supplementary file 7 — Source data [file 41467_2019_13960_MOESM7_ESM.zip › Source_Data/Figure6/Figure6B_MotifScanOutput/ETS1_mutPU.1/homerResults/motif11.similar.html]

motif11

## Information for motif11

C
T
A
G
A
G
T
C
C
A
G
T
A
C
T
G
G
C
A
T
G
A
C
T
C
G
T
A
C
T
G
A
  
Reverse Opposite:  

G
A
C
T
G
C
A
T
C
T
G
A
C
G
T
A
T
G
A
C
G
T
C
A
T
C
A
G
G
A
T
C
  

|  |  |
| --- | --- |
| p-value: | 1e-150 |
| log p-value: | -3.461e+02 |
| Information Content per bp: | 1.540 |
| Number of Target Sequences with motif | 8758.0 |
| Percentage of Target Sequences with motif | 64.95% |
| Number of Background Sequences with motif | 18197.5 |
| Percentage of Background Sequences with motif | 51.86% |
| Average Position of motif in Targets | 100.5 +/- 56.4bp |
| Average Position of motif in Background | 100.8 +/- 64.3bp |
| Strand Bias (log2 ratio + to - strand density) | -0.0 |
| Multiplicity (# of sites on avg that occur together) | 1.53 |
| Motif File: | file (matrix) reverse opposite |

### Similar de novo motifs found

|  |  |  |  |  |  |  |  |
| --- | --- | --- | --- | --- | --- | --- | --- |
| Rank | Match Score | Redundant Motif | P-value | log P-value | % of Targets | % of Background | Motif file |
| 1 | 0.965 | C T A G A G T C G C A T A C T G G C A T A G C T C G T A | 1e-106 | -246.361907 | 49.45% | 38.42% | motif file (matrix) |
| 2 | 0.899 | G A T C C T A G A C T G T G A C C G A T A C T G G A C T G A C T C G T A T C G A | 1e-65 | -149.855018 | 16.46% | 10.64% | motif file (matrix) |
